# Early clinical experiences with AI-based EVAR planning using the Endoleak Risk Index support its value for individualized decision-making and education

**DOI:** 10.1016/j.jvscit.2025.102037

**Published:** 2025-10-30

**Authors:** Paula Rosalie Keschenau, Mats Döring, Sharif Elshafei, Daniel Palacios, Mirja Stark, Mohammed Ghazal, Jean-Noël Albertini, Johannes Kalder

**Affiliations:** a Justus Liebig University Giessen, Faculty 11, Department of Adult and Pediatric Cardiovascular Surgery, University Hospital Giessen, Giessen, Germany; b Department of Vascular and Endovascular Surgery, Saint-Joseph Hospital, Marseilles, France

**Keywords:** Artificial intelligence, Digital twin, Endoleak, Endovascular aortic repair

## Abstract

**Objective:**

The aim of this study was to evaluate our first experience with the use of the artificial intelligence-based Endoleak Risk Index (ERI) in the planning of infrarenal endovascular aortic repair with special regard on its impact on clinical decision-making.

**Methods:**

This single-center study evaluated two patient groups treated with Endurant endovascular aortic repair (EVAR). Group 1 comprised a retrospective cohort with at least 3 years of follow-up. The ERI was calculated for this group from preoperative computed tomography angiography scans and compared with the actual outcome. The prospective group 2 included patients scheduled for elective EVAR from March 2024 to March 2025, with preoperative AI-based simulations for endograft sizing including ERI calculation for type 1a endoleak (EL1a) risk prediction. The influence of ERI on clinical decision-making was assessed. Patients with noncontrast computed tomography scans or scans with slice thickness greater than 3 mm were excluded.

**Results:**

Twenty patients were included, with 10 in each group and a median age of 70 years (range, 59-81 years) in group 1 and 72 years (range, 60-84 years) in group 2. In group 1, the ERI was elevated in six of 10 cases, with four patients experiencing perioperative or late EL1as during follow-up. Notably, two patients with elevated ERI did not develop EL1a over follow-up periods of 93 and 44 months, whereas all patients with low ERI remained free of endoleaks. Two patients in group 2 had low ERI and no EL1a, whereas two had elevated ERI for at least one simulated endograft size, leading to a change in treatment (larger endograft) for one patient. The remaining six patients had elevated ERI for all simulated sizes, with one case being unsuitable for infrarenal EVAR. Despite elevated ERI, the initial treatment plan remained unchanged for four patients, one of whom died due to cardiac reasons before implantation. Overall, no patients in group 2 developed EL1a during a median follow-up of 3 months (range, 1-12 months).

**Conclusions:**

This pilot study suggests that ERI calculation can be valuable even in straightforward cases, emphasizing the importance of education in the EVAR planning process. With further validation from larger datasets and advancements in technology, artificial intelligence-based EL1a risk prediction has the potential to significantly enhance EVAR planning in the future, promoting patient safety by providing tailored treatment strategies and supporting the surgeon in his decision-making.

Artificial intelligence (AI)-based technologies are evolving rapidly, and numerous medical applications are appearing on the market, also in the field of vascular surgery.^1^^,^^2^ In abdominal aortic aneurysm (AAA) care alone, several applications of AI-based solutions are being developed and explored, among others for automated AAA detection and size measurement as well as growth, rupture risk, and complication prediction.^3^

Another interesting approach is the use of AI-based software for supporting treatment planning, namely in endovascular procedures. The AI-based digital-twin technology for patient-specific planning of endovascular aortic repair (EVAR) (PrediSurge), has emerged as one of the most promising technologies in this regard. Taking into account the patient’s individual aorto-iliac anatomy and biomechanical properties of both human aortic tissue and the stent graft planned for implantation, it allows a preoperative case-specific EVAR simulation.^4^

Based on this technology, a risk index predicting the risk for a postoperative type 1 a endoleak (EL1a) has been developed and has gained CE-certification for the Endurant endograft (Medtronic Endovascular) recently as the so-called Endoleak Risk Index (ERI). Although the risk factors for EL1a are supposedly well-known, having been the subject for research for decades,^5^, ^6^, ^7^, ^8^, ^9^ they still constitute a clinically relevant problem contributing to secondary ruptures and increased long-term mortality after EVAR.^10^^,^^11^ Recently, the estimated freedom from EL1a 6 years after elective EVAR has been reported to be 76%.^12^ Therefore, the ERI could be a useful tool to support EVAR planning with the goal of improving patient outcomes. However, as the ERI is a very young technology that is only just being introduced into medical practice, its actual usefulness in the clinical setting has yet to be evaluated.

Therefore, the aim of this study was to evaluate our first experience with the use of the AI-based ERI in the planning of infrarenal EVAR with special regard to its impact on clinical decision-making and education. This pilot study was meant to provide first insights into the usefulness of implementing this new technology into clinical routine and give starting points for future research.

## Methods

The objective of this pilot study was, after evaluating the correctness of AI-based EL1a prediction in a retrospective patient cohort at our own center, to gain first insights into the effect preoperative EVAR simulation on clinical decision-making with change of treatment plan being the primary outcome and the EL1a being a secondary outcome. Therefore, a two-cohort design was chosen for this single-center study, including two groups of patients who were treated with Endurant (Medtronic Endovascular) EVAR.

Group 1 was a historic cohort of patients who underwent Endurant EVAR in the past using conventional planning and had a follow-up of at least 3 years so that the outcome regarding the occurrence of an early or late EL1a was known. Patient data in group 1 were gathered retrospectively. In this group, the ERI (PrediSurge SAS) was calculated retrospectively based on the preoperative computed tomography angiography (CTA) scan and the implanted endograft size. In addition, because the original measurements were unavailable, standard measurements including aortic neck diameter, length, and angulation were redone so as to be able to describe the neck morphology. The prediction of the EL1a risk using the ERI was compared with the actual outcome, including intraoperative results up to the end of follow-up. Additionally, although bearing in mind the limitation due to the small cohort, a Kaplan-Meier analysis was performed for this cohort in order to illustrate potential time-to-event trends regarding EL1a occurrence.

Group 2 consisted of all patients planned for an elective infrarenal EVAR between March 2024 and March 2025 with preoperative AI-based simulation of the planned Endurant endograft size or sizes. Nonelective patients, patients with other infrarenal endografts than Endurant, and patients without preoperative AI-based simulation (eg, due to insufficient imaging quality) were excluded. Patient data in group 2 were collected prospectively and analyzed retrospectively. Initial endograft planning was performed by the operating vascular surgeon based on three-dimensional multiplanar reconstructions of the preoperative CTA scan as recommended in the recent clinical practice guidelines of the European Society of Vascular Surgery.^13^ Then, the simulation results were considered, and the final treatment plan was made. This final treatment decision was always made by the operating surgeon and supported by a team discussion. In this discussion, standard parameters of the infrarenal neck like diameter and diameter change, thrombus burden, calcifications, angulation, and length were assessed together, giving most weight to the opinion of the most experienced endovascular surgeons. In addition to comparing the ERI results with the outcome regarding EL1a occurrence during early follow-up (FU), the impact of the ERI result on the clinical decision making was recorded. FU examinations were performed according to the clinical routine with duplex ultrasound and/or CTA scans. According to the reporting standards for EVAR^14^ the time of events after EVAR implantation, in particular EL1a occurrence, was defined as follows: perioperative as within 30 days of implantation, early as 30 days to <6 months, mid-term as 6 months to <5 years, and long-term as 5 years or more.

In both groups, patients who had noncontrast-enhanced CT scans or CTA scans with a slice thickness >3 mm were excluded. The study was approved by the Institutional Ethics Committee of the Medical Faculty of the Justus-Liebig-University Giessen (AZ132/21). The statistical analyses were performed using Microsoft Excel 2019 (Microsoft Corporation) and IBM SPSS statistics 25.0 (2017, IBM Corporation).

### The Endoleak Risk Index

The ERI, based on the Digital Twin Technology (PrediSurge SAS), is a commercially available and CE-certified software solution for predicting the EL1a before EVAR implantation. Using finite-element analysis, a patient-specific digital twin of the aorta is generated based on the preoperative CTA scan integrating biomechanical properties of the aortic tissue. Then, similarly, a digital twin of the Endurant stent graft is generated integrating the material properties, and the stent graft deployment in the patient-specific digital aortic twin is simulated. This digital twin technology and its use for prediction of proximal sealing in EVAR including development of the ERI have been described in detail by Derycke et al and Albertini et al.^4^^,^^15^

Briefly, the ERI is calculated based on a detailed analysis of the proximal landing zone from about 50 indicators that are chosen using feature engineering and combined into a single risk index using machine learning algorithms. Tens of thousands of data points per patient were the basis for developing the indicators that include, among other circularity indices, apposition indices of endograft against the aorta, diameter, oversizing, and many others.^4^^,^^15^ The ERI is designed as a binary value (elevated risk or low risk) and given with a confidence level. In the beginning, the confidence level was given on the simulation report as “high” or “low”; now it is given as (“highly likely” *P* ≥ 75%, “likely” 50% < *P* < 75%, “unlikely” 25% < *P* < 50% or “very unlikely” *P* ≤ 25%).

## Results

**Fig 1:**
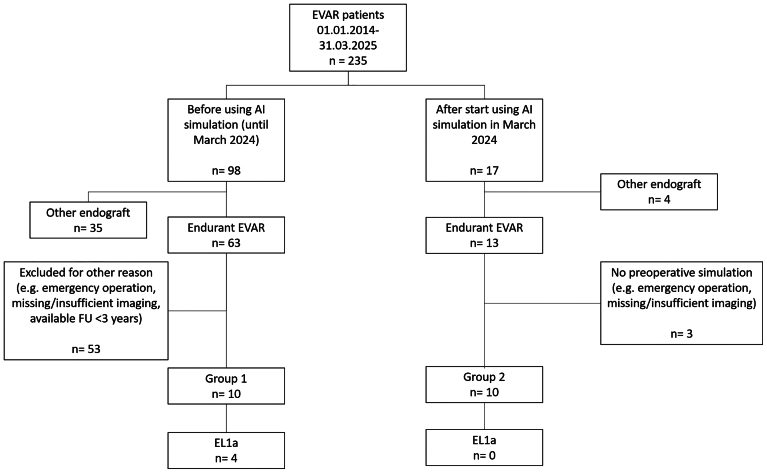
Flow chart demonstrating patient inclusion/exclusion in groups 1 and 2. *AI*, Artificial intelligence; *EL1a*, type 1a endoleak; *EVAR*, endovascular aortic repair; *FU*, follow-up.

Overall, 20 patients were included in this study, 10 belonging to group 1 and 10 to group 2 (Fig 1). The median patient age was 70 years (range, 59-81 years) in group 1 and 72 years (range, 60-84 years) in group 2. Table I gives an overview over basic patient characteristics in both groups.

**Table I:** Basic patient characteristics in groups 1 and 2

	Group 1 (n = 10)	Group 2 (n = 10)
Age, years	70 (59-81)	72 (60-84)
Height, cm	175 (163-187)	179 (171-182)
Weight, kg	76 (62-107)	93 (76-100)
BMI, kg/m^2^	27 (23-40)	30 (25-32)
Male sex	9	10
Coronary artery disease	7	7
Prior myocardial infarction	2	1
Peripheral arterial disease	2	1
Diabetes mellitus	4	3
Arterial hypertension	10	9
Cerebrovascular disease/prior stroke	2	1
Chronic renal insufficiency	1	1
Hyperlipidemia	7	4
Chronic obstructive pulmonary disease	0	4

*BMI,* Body mass index.

Data are presented as number or median (range).

**Fig 2:**
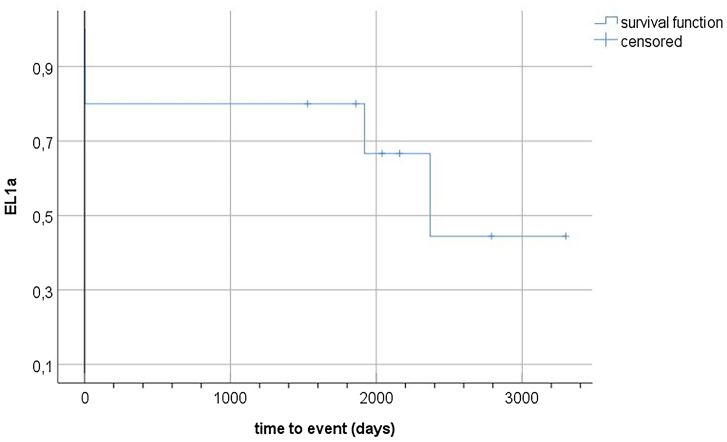
Kaplan-Meier curve illustrating potential time-to-event trends for type 1a endoleak (*EL1a*) occurrence in group 2.

In group 1, the ERI was elevated in six of 10 cases. Four patients with elevated ERI developed an EL1a perioperatively or during late FU (n = 2, respectively). Both patients with perioperative EL1a were treated endovascularly at <30 days after the initial procedure. One of the patients with late EL1a underwent open surgical conversion; the other refused therapy due to advanced age and comorbidity. Two patients had no EL1a despite an elevated ERI during a FU of 93 and 44 months. All patients (4/10) with low EL1a risk according to the ERI remained without endoleak during mid and long-term FU. The outcomes of group 1 regarding EL1a are depicted in Table II. Additionally, the Kaplan-Meier curve presented in Fig 2 illustrates potential time-to-event trends regarding EL1a occurrence in group 1. Other reinterventions in group 1 were distal extensions in three cases and open surgical ligation or coil embolization of the inferior mesenteric artery (n = 1, respectively).

**Table II:** Results of the Endoleak Risk Index (*ERI*) in the retrospective patient cohort (group 1)

Patient	Diameter proximal neck, mm (in 5-mm steps starting from lowest renal artery)^a^	Neck length (diameter variation <10%)^a^	Infrarenal angle^a^	Maximum AAA size, mm^a^	Recommended EVAR size/sizes (as per Medtronic sizing sheet)	Implanted Endurant main body	ERI	EL1a	Time from surgery to event^b^
1	19-19-20-19-20	35	10	83	23	ETBF2516C124EE	**0.7454**	No	93 months
2	19-20-21-22-21	42	17	80	23/25	ETBF2516C166EE	*0.42*	No	110 months
3	20-21-24	12	24	48	23/25/28	ETBF2816C166EE	**0.8743**	Yes, late	64 months
4	26-28-29-30	13	53	75	32/36	ETBF3216C145EE	**0.9316**	Yes, perioperative	4 days
5	21-21-24	12	36	64	25/28	ETBF2816C145EE	**0.93**	Yes, late	79 months
6	23-24-24-26-26	39	42	89	28/32	ETBF2516C166EE	**0.8698**	Yes, perioperative	5 days
7	23-21-21-25	21	63	56	25/28	ETBF2516C145EE	**0.92**	No	68 months
8	21-20-22-20-21	26	26	51	23/25	ESBF25114C103EE	*0.09*	No	51 months
9	25-24-23-24-25	44	10	52	28	ETBF2816C145EE	*0.18*	No	72 months
10	21-22-23-25-24-24	25	9	51	25/28	ETBF2816C166EE	*0.47*	No	62 months

*AAA,* Abdominal aortic aneurysm; *EL1a,* type 1a endoleak; *EVAR,* endovascular aortic repair.

Recommended EVAR sizes as per Medtronic sizing chart are given for all diameters measured along the aortic neck.

The ERI is given as a binary value value (**elevated risk** or *low risk*) with a confidence level (“highly likely”: *P* ≥ .75, “likely“ .5 < *P* < .75, “unlikely“ .25 < *P* < .5% or “very unlikely” *P* ≤ .25).

Bold values, elevated risk; Italic values, low risk.

a Retrospective re-measurement of all cases.

b Event = date of first diagnosis of EL1a or last follow-up without EL1a.

In group 2, the ERI was low in two of 10 cases; the initially planned EVAR size was maintained, and both patients had no EL 1a during the further course.

In three of 10 patients, the ERI was elevated for at least one of the simulated endograft sizes. In two of those patients (patients 3 and 4), both simulated EVAR sizes could have been chosen based on the sizing chart recommendations, yet the smaller one showed an elevated ERI. Because the initially planned EVAR size was already the larger one, this treatment decision was maintained, supported by the low ERI. In the third patient (patient 8), simulation of the smaller EVAR size was proposed by a junior team member due to differing measurements, and the resulting ERI was elevated for this smaller size. The initial treatment decision of the larger EVAR size made by the other (senior) team members was maintained, supported by the simulation result.

The remaining five patients in group 2 had elevated ERI for all simulated endograft sizes. In one of those cases (patient 6), with an unfavorable neck anatomy, EVAR was discarded as a treatment option and a custom made fenestrated endograft was ordered. At the same time, this was the only case that would have been outside the Endurant instructions for use (IFU); all other EVAR cases were within the Endurant IFU. In another patient (patient 10), a larger endograft than planned was chosen due to the simulation result and subsequent team discussion.

The initial treatment plan was not changed despite the elevated ERI in four patients. One of them, however, died due to cardiac cause before EVAR implantation. Further, in one patient, only one endograft size had been simulated preoperatively. This was the first patient in this group, and the operating surgeon changed at short notice. Interestingly, without knowing the simulation result, the new surgeon chose an endograft from a different manufacturer but of the same size as the initially planned one.^11^ The patient remained free from an EL1a during a FU of 12 months. Overall, none of the patients in group 2 developed an EL1a during a median FU of 3 months (range, 1-12 months) so far. The results of group 2 are depicted in detail in Table III.

**Table III:** Results of the Endoleak risk index (*ERI*) in the prospective patient cohort (group 2) and impact on treatment decision

Patient	Age, years	Simulation result (top row: ERI for planned EVAR size, second row: one size larger,if applicable, third row: one size smaller, if applicable)	Diameter proximal neck, mm (in 5-mm steps starting from lowest renal artery)	Neck length (diameter variation <10%)	Infrarenal angle	Recommended EVAR size(s) (as per Medtronic sizing sheet)^a^	Size of planned EVAR	Inside IFU	Change of treatment plan based on simulation result	Size of implanted EVAR	EL1a	Duration of FU, months
1	80	ETBF2813C124EE **ERI high**	24-24-25-25	20	55	28	28	Yes	No^b^	28	No	12
2	84	ESBF3614C103EE **|ERI = 0.7152**	28-29-30-29-27	71	57	32/36	36	Yes	No	NA	NA	NA
		ESBF3214C103EE **|ERI = 0.7321**										
3	80	ESBF3214C103EE **|ERI = 0.18**	29-25-23-22	15	25	25/28/36	32	Yes	No	32		6
		ESBF3614C103EE **|ERI = 0.1294**										
		ESBF2814C103EE **|ERI = 0.7882**										
4	76	ETBF3216C124EE **|ERI = 0.3436**	23-25-28-25	30	18	28/32	32	Yes		32	No	6
		ETBF2816C124EE **|ERI = 0.8685**										
5	72	ESBF2314C103EE **|ERI = 0.1871**	21-20-19-20	37	4	23/25	23	Yes	No	23	No	3
		ESBF2514C103EE **|ERI = 0.0864**										
6	69	ETBF3216C145EE **|ERI = 0.9304**	24-25-27	9	0	28/32	32	No	Yes, fenestrated EVAR planning	NA	NA	NA
		ETBF3616C145EE |**ERI = 0.9132**										
		ETBF2816C145EE **|ERI = 0.9291**										
7	60	ESBF2814C103EE **|ERI = 0.2057**	25-22-21	20	9	25/28	28	Yes	No	28	No	3
		ESBF3214C103EE **|ERI = 0.1651**										
8	60	ESBF2814C103EE **|ERI = 0.3163**	24-22-23	18	6	28	28	Yes	No	28	No	2
		ESBF2514C103EE **|ERI = 0.8286**										
9	71	ESBF3214C103EE **|ERI = 0.901**	27-27-26-29	20	36	32/36	32	Yes	No	32	No	1
		ESBF3614C103EE **|ERI = 0.7607**										
10	72	ESBF3214C103EE **|ERI = 0.8817**	26-27-28	10	60	32	32	Yes	Yes, larger endograft	36	No	1
		ESBF3614C103EE **|ERI = 0.6611**										
		ESBF3214C103EE **|ERI = 0.8817**										

*El1a,* Type 1a endoleak; *EVAR,* endovascular aortic repair; *FU,* follow-up; *IFU,* instructions for use; *NA,* not applicable.

Simulation results are presented as in the clinical workflow: top row – ERI for planned EVAR size after standard sizing; second row – ERI for EVAR one size larger (if applica-ble); third row – ERI for EVAR one size smaller (if applicable).

Recommended EVAR sizes as per Medtronic sizing chart are given for all diameters measured along the aortic neck.

The ERI is given as a binary value (elevated risk or *low risk*) with a confidence level (“highly likely”: *P* ≥ .75, “likely“ .5 < *P* < .75, “unlikely“ .25 < *P* < .5% or “very unlikely“ *P* ≤ .25).

Bold values, elevated risk; Italic values, low risk.

a Recommended sizes for the diameters measured along the aortic neck.

b Other manufacturer based on surgeon’s preference without knowing simulation result.

## Discussion

This study describes a first single-center experience with AI-based EL1a risk prediction using the digital-twin technology to support infrarenal EVAR planning in clinical routine. It combines a retrospective control group from the same institution for internal validation of industry-given data on sensitivity and specificity of the ERI with a prospective patient cohort demonstrating favorable early clinical experiences. These first results can serve as a basis to conduct further larger-scale studies with longer FU to draw final, statistically robust conclusions.

The results of the retrospective cohort of the present study show that an early or late EL1a was predicted correctly in all cases by an elevated ERI, and the ERI was low in all cases without EL1a during mid and long-term FU. Two of 10 false positives were observed in the present own patient collective, so that our results are in accordance with the current industry-given sensitivity and specificity of the ERI for EL1a detection of 96% and 78%, respectively.^1^ Acknowledging the limited patient number, the concordance of our own observation with these data from the first retrospective ERI validation studies supported us in introducing the ERI calculation on a routine-basis into our clinical practice so as to gain first clinical experiences.

Naturally, due to the early stages of this technology, basing treatment decisions on the ERI alone cannot yet be recommended. Therefore, all prospective cases were also measured and planned as per our clinical routine, which is in accordance with the current European Society of Vascular Surgery EVAR planning recommendations (use of thin-slice CTA scans and multiplanar reconstructions).^13^ Then, the results of the ERI were taken into consideration. The cases with a positive ERI were evaluated critically by discussing them in our vascular team. Based on the team discussion, the initial plan was maintained in three of five cases with positive ERI for the planned endograft size and changed in the remaining two. In patient 10 of group 2, a larger endograft size was chosen based on the simulation result, which may correspond to the “gut feeling” of experienced endovascular surgeons. Indeed, it has also been demonstrated in the literature that the more unfavorable neck characteristics come together, the higher the risk for an EL1a.^16^ However, despite the recommendations for oversizing in EVAR being well-known, a relevant proportion of patients with EL1a after EVAR are insufficiently oversized.^17^ Thus, there seems still to be a problem in this regard in clinical practice, for which the preoperative simulation and ERI calculation might be of help. On the other hand, it has to be pointed out that increased oversizing is not always the best option. Simulation and ERI results should be interpreted with care and, in case of an elevated ERI, the possibility of a false positive result should be kept in mind, and different treatment options should be considered on a patient-individual basis (eg, smaller endograft size plus primary endoanchor implantation, fenestrated EVAR, or discarding endovascular therapy and opting for open repair).

**Fig 3:**
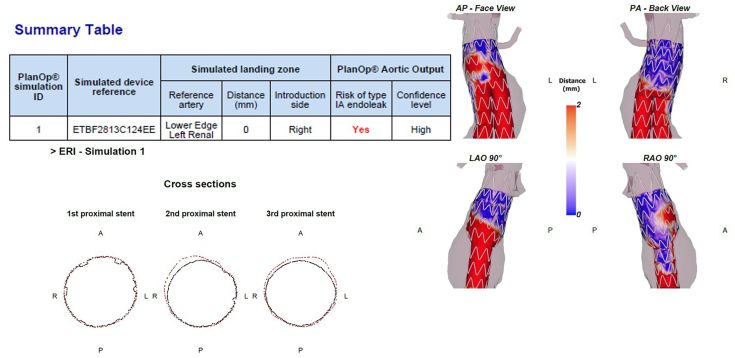
Preoperative simulation result of patient 1 in group 2 showing suboptimal apposition of the planned 28 mm Endurant stent graft in the second and third stent rows in the distance color map as well as in the cross sections.

**Fig 4:**
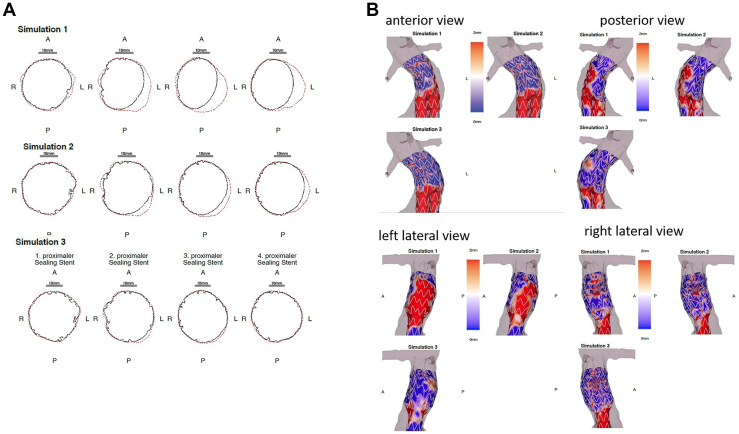
Preoperative simulation result of patient 10 in group 2 of three endograft sizes. The cross sections **(A)** as well as the color maps **(B)** show very nicely the lack of apposition in the proximal sealing zone for an undersized graft (Simulation 1: 28 mm) but also for a properly sized graft (Simulation 2: 32 mm). Choosing more oversizing (Simulation 3: 36 mm), the apposition improves but there might be a risk of infolding in the first and maybe second stent row. In this case, the simulation result prompted choosing a larger oversizing with a good early result. *A*, Anterior; *L*, left; *P*, posterior; *R*, right

Another relevant finding of our first clinical experiences with digital simulation in EVAR planning was that not only the simulation and ERI results themselves but also the intensified team discussions, which were of great educational value for residents and younger vascular surgeons with little endovascular experience. This became most apparent in case 6, with a short conical neck that maybe should not have been considered for EVAR in the first place, and in the very first case, where one might argue that a conical neck has been overlooked (Fig 3). Anticipating endograft behavior during the planning process becomes easier with increasing experience, but the learning curve might be accelerated by using AI support in EVAR planning. The three-dimensional visualization by the preoperative AI-based EVAR simulation can stimulate team discussion by appraising different treatment options. The distance color maps help to illustrate areas of insufficient apposition (Figs 3 and 4), and the cross sections visualize the EL1a risk due to lack of apposition of a smaller graft vs infolding risk with larger oversizing (Fig 4). Further, despite the operator’s experience being highly relevant, planning mistakes can always happen, especially considering the time pressure in everyday clinical practice. Therefore, we found that “being alerted” to supposedly easy cases by a positive ERI is quite useful even if, considering the current ERI sensitivity of 78% that is supported by our own experience in the retrospective patient cohort, false-positive results are also to be expected. Long-term FU will show whether false-positive results explain the elevated ERI of some patients of group 2. Moreover, investigating the benefit of secondary case review with or without additional digital simulation could be of interest for further studies on EVAR planning.

Taking these considerations together, another potential benefit of the preoperative EVAR simulation lies in reinforcing decision confidence, particularly for less experienced surgeons and trainees. As demonstrated by several cases of both groups, one might be unsure which endograft size is optimal when the measured diameters correspond to different recommended EVAR sizes according to the manufacturer’s sizing chart. It can be helpful to be assured about the treatment decision, and higher confidence may potentially contribute to better performance. In general, using new technologies (eg, simulator training and virtual and augmented reality) to improve surgeon education and training is being established more and more in different surgical specialties due to the obvious benefits.^18^^,^^19^ Discussing all new educational approaches would be beyond the scope of this manuscript, but based on our experiences and feedback from junior team members and trainees so far, we believe that preoperative AI-based EVAR simulation can contribute significantly to endovascular surgical training.

Looking at the mean patient age in both cohorts, another potential benefit of AI-supported EVAR planning comes to mind. Currently, the ERI does not differentiate between early and late EL1a. Should this be possible in the future, it may help to guide individualized decision-making for elderly patients who may benefit from accepting EVAR in a suboptimal neck anatomy when the EL1a is only expected in the long-term.

Moreover, it has to be pointed out that the proximal sealing zone and EL1a risk are only one part contributing to long-term EVAR success. Thus, the ERI is just one part of the puzzle, and there are many future directions that should be explored and questions to be addressed regarding AI-supported predictive EVAR-planning (ie, distal landing zone assessment and type 2 endoleaks).^2^^,^^4^

### Study limitations and future perspectives

This pilot study included mostly standard neck anatomies within the Endurant IFU. It may be argued whether routine preoperative AI-based simulation and ERI calculation is really necessary and economically reasonable or should maybe be reserved for selected difficult neck anatomies. However, despite the small absolute number of cases—explainable by inclusion/exclusion criteria and the fact that our tertiary care center focuses on more complex juxta-/suprarenal, thoracic, and thoracoabdominal aortic pathologies—as well as the only partial view of clinical outcomes in the prospective group 2 due to short FU and two cases remaining without EVAR implantation (due to unexpected death and treatment change to fenestrated EVAR), we found that the ERI calculation was useful also in supposedly straightforward cases. In our opinion, based on these positive first clinical experiences and provided further validation in larger retrospective and prospective data sets as well as further developments, this technology has the potential to become a key part of future EVAR planning.

It has to be kept in mind that when assessing early-phase innovations such as the ERI, pilot studies like the present one are required to justify investing in a novel product and setting up well-designed and statistically sound larger scale investigations so as to gain meaningful conclusions in the end. Thus, the results and conclusions drawn from this small-scale study can serve as basis for future research on the usefulness of AI-based EVAR planning using the digital twin technology regarding EL1a risk prediction, complication reduction, and individualization of therapy, as well as educational and cost-effectiveness aspects.

All in all, it is to be assumed that the combination of different AI-based solutions, including preoperative diagnostics, operation planning tools, and intraoperative support and guidance, as well as postoperative surveillance tools will significantly change the current EVAR practice in the future.^20^, ^21^, ^22^ However, at least today, the final decision and responsibility still lies with the implanting surgeon who has to appraise the results of supporting AI tools thoughtfully.

## Conclusions

Provided there is further validation on larger retrospective and prospective data sets as well as further development of this new technology, AI-based EL1a risk prediction for EVAR using the ERI has the potential to significantly enhance EVAR planning in the future. It may help improve patient security by individualizing treatment strategies on one hand and education on the other hand. The final decision and responsibility, however, still lie with the implanting surgeon.

## Funding

None.

## Disclosures

P.R.K. and J.K. are consultants for PrediSurge and Medtronic. J.N.A. is co-founder of PrediSurge SAS. The remaining authors report no conflicts.
